# Supplementary material for: Intensity of de novo DSA detected by Immucor Lifecodes assay and C3d fixing antibodies are not predictive of subclinical ABMR after Kidney Transplantation
Source: PLoS One. 2021 Apr 22;16(4):e0249934. doi: 10.1371/journal.pone.0249934 (PMC8062066; doi:10.1371/journal.pone.0249934)
Supplement: Attachment — Submitted filename: Review Comments to the Author_Intensity of de novo DSA detected by Immucor Lifecodes Assay and C3d fixing antibodies are not predictive of subclinical ABMR after Kidney Transplantation.pdf [file pone.0249934.s001.pdf]

Review Comments to the Authors
Manuscript title: Intensity of de novo DSA detected by Immucor Lifecodes Assay and C3d fixing antibodies are not predictive of subclinical ABMR after Kidney Transplantation.

The rationale and objective of this study is clear. Moreover, authors successfully describe the contribution of this work to the field, while recognizing its limitations and noting outstanding questions. However, the manuscript requires much improvement to the writing quality and data presentation and interpretation. The manuscript lacks organization and focus due to run-on and awkwardly structured sentences. In addition, figures are missing crucial details required to interpret the data. Several results are stated without a clear connection to the supporting data.

Authors should address the following points:

- Readers could benefit from a more detailed description of HLA donor specific antibodies
- and de novo DSA in the introduction, and how this is relevant to transplant pathology.
- Please explain rationale for accepting positive results for the IM Lifecodes LSAB after 2 of 3 criteria were met, instead of all requiring all criteria to be met.
- Please indicate negative control beads for the IM Lifecodes LSAB.
- Authors should specify concentration of beads, phycoerythrin conjugated goat antihuman IgG, anti-human C3d, instead of, or in addition to the volume used, as volume is arbitrary without knowing this information.
- Please describe methods for light microscopy and immunofluorescence staining of kidney tissue.
- In statistical analysis, authors should indicate a Student t-test was used.
- Please describe the advantages/disadvantages of the iDSA and DSA parameters. For example, why were both used, and is one superior to the other?
- Please indicate all abbreviations used in Table 1.
- Authors should consider reformatting the methods and results sections from bullet points to paragraphs.
- For reader's ease, authors should consider labeling Figure 1 panels A-C to indicate the correlation described (as indicated in the Figure legend).
- For Figure 2, please define the descriptive statistics reflected in the box and whisker plot.
- Please label y-axes in Figures 2, 3, 4, 5, and 6.
- Please specific what the numbers refer to on the top of each bar in Figure 3. In addition, please explain in more detail the significance of Figure 3.
- Figure 4 is not clearly described and thus, difficult to interpret. To address this, please indicate the abbreviations used (g+cpt, i+t, ci+ct, cv, and cg) in the figure legend. Please define the numbers indicated on top of the bar graphs in the figure legend.
- Authors should indicate the Figure showing the data supporting the following results: "The proportions of patients with positive C3d-fixing DSA were not statistically different in patients with active sABMR, chronic active sABMR or without sABMR (56.7% vs 47.1 % vs 57.1%, p=NS)."
- Please indicate the figure showing the data supporting the following results that begin with the sentence: "The intensity (BCM) of iDSA and sDSA were statistically higher in the C3d-fixing DSA group…"
- Please be consistent with reporting non-significant results as either "NS" (without a p-value) or reporting a p-value greater than 0.05. Preference is to report p-values. Authors should also consider reporting actual p-values for significant data (instead of indicating p<0.0001).
- This sentence is awkwardly written: "In our previous published study 4, we demonstrated that this strategy led to the diagnosis of sABMR in up to 40 %."

- The discussion should be condensed to be more concise and direct because it lacks clear focus. The discussion should also better delineate between the findings from the group's previous work and the findings form the current study. Finally, authors should be cautious about describing data as "perfectly comparable" to previous studies.
- "Finally" is spelled incorrectly in the discussion.
