# Supplementary material for: Intensity of de novo DSA detected by Immucor Lifecodes assay and C3d fixing antibodies are not predictive of subclinical ABMR after Kidney Transplantation
Source: PLoS One. 2021 Apr 22;16(4):e0249934. doi: 10.1371/journal.pone.0249934 (PMC8062066; doi:10.1371/journal.pone.0249934)
Supplement: Attachment — Submitted filename: Response to Reviewers.docx [file pone.0249934.s002.docx]

The study examined the role of de novo subclinical DSA and their potential C3-fxing antibody. Based on over 100 patients, the authors conclude that no correlation was observed between subclinical antibody mediated rejection (sAMR) and the detection of C3-fixing antibody.

**The reviewer # 1 requested that the authors make more conclusive statement based on their analysis. Indeed, the statement that more study should be performed is not satisfactory as almost every study needs a continuation.**

Thank you for your analysis. We tried to make more conclusive statement in our revised manuscript.

**The Reviewer # 2 indicated at several editorial changes which need to be addressed.**

**• Readers could benefit from a more detailed description of HLA donor specific antibodies**

We added in the introduction a more detailed description on HLA DSA and dn DSA

**• and de novo DSA in the introduction, and how this is relevant to transplant pathology.**

We added in the introduction a more detailed description on HLA DSA and dn DSA.

• **Please explain rationale for accepting positive results for the IM Lifecodes LSAB after 2 of 3 criteria were met, instead of all requiring all criteria to be met.**

Thank you for your comment. Accepting positive results for the IM Lifecodes LSAB after 2 of 3 criteria were met is used according to the manufacturers' instructions. Nevertheless, when BCM is low (below 1500) but the amount of antigen in the bead is also low, result could be positive because BCR and AD-BCR are positive.

**• Please indicate negative control beads for the IM Lifecodes LSAB.**

As indicated in the methods section, the negative control beads typically yield low MFI values (lot specific) when treated with the positive or negative control sera.

**• Authors should specify concentration of beads, phycoerythrin conjugated goat antihuman IgG, anti-human C3d, instead of, or in addition to the volume used, as volume is arbitrary without knowing this information.**

We completely understand your comment. Unfortunately, all these components are provided with LIFECODES LSAB and LIFECODES C3d kits by the manufacturer and the concentration of beads, phycoerythrin conjugated goat antihuman IgG, anti-human C3d are not available.

**• Please describe methods for light microscopy and immunofluorescence staining of kidney tissue.**

Thank you for your comment. Light microscopy analysis was performed after fixation in Dubosq Brazil solution, 2-μm paraffin section, and coloration by Masson trichrome, hematoxylin & eosin, periodic acid-Schiff and Marinozzi silver stainings. C4d staining was performed on paraffin sections by immunohistochemical (IHC) analysis using a rabbit anti-human C4d polyclonal IgG, or by immunofluorescent staining performed on 4-μm frozen sections with monoclonal mouse anti-human C4d antibody. We added this point in the revised manuscript.

**• In statistical analysis, authors should indicate a Student t-test was used.**

This point was added in the statistical analysis section.

**• Please describe the advantages/disadvantages of the iDSA and DSA parameters. For example, why were both used, and is one superior to the other?**

Thank you for your comment. Both iDSA and sDSA were used in this study because some kidney transplant recipients had more than 1 DSA. Even if the intensity of the iDSA is not sufficient to predict subclinical ABMR, the combination of all DSA would be.

Réf: Bertrand D, Farce F, Laurent C, et al. Comparison of Two Luminex Single-antigen Bead Flow Cytometry Assays for Detection of Donor-specific Antibodies after Renal Transplantation. Transplantation. 2019;103(3):597–603. doi:10.1097/TP.0000000000002351

**• Please indicate all abbreviations used in Table 1.**

We indicate all abbreviations used in Table 1 in the revised manuscript.

**• Authors should consider reformatting the methods and results sections from bullet points to paragraphs.**

Thank you for your help to improve the methods and results sections, presented in the revised manuscript.

**• For reader’s ease, authors should consider labeling Figure 1 panels A-C to indicate the correlation described (as indicated in the Figure legend).**

We corrected this point in the manuscript

**• For Figure 2, please define the descriptive statistics reflected in the box and whisker plot.**

We defined the descriptive statistics reflected in the box and whisker plot.

**• Please label y-axes in Figures 2, 3, 4, 5, and 6.**

We labeled y-axes in Figures 2, 3, 4, 5, and 6.

**• Please specificy what the numbers refer to on the top of each bar in Figure 3. In addition, please explain in more detail the significance of Figure 3.**

Thank you for your comment. We made these corrections in the revised manuscript.

**• Figure 4 is not clearly described and thus, difficult to interpret. To address this, please indicate the abbreviations used (g+cpt, i+t, ci+ct, cv, and cg) in the figure legend. Please define the numbers indicated on top of the bar graphs in the figure legend.**

Thank you for your comment. We made these corrections in the revised manuscript.

**• Authors should indicate the Figure showing the data supporting the following results: “The proportions of patients with positive C3d-fixing DSA were not statistically different in patients with active sABMR, chronic active sABMR or without sABMR (56.7% vs 47.1 % vs 57.1%, p=NS).”**

Thank you for your comment. We did not add an extra figure for this point meaning that it was not useful for the readers.

**• Please indicate the figure showing the data supporting the following results that begin with the sentence: “The intensity (BCM) of iDSA and sDSA were statistically higher in the C3dfixing DSA group…”**

Thank you for your comment. Figure 7 about the intensity (BCM) of iDSA and sDSA according C3d fixing DSA positivity was added in the revised manuscript.

**• Please be consistent with reporting non-significant results as either “NS” (without a p-value) or reporting a p-value greater than 0.05. Preference is to report p-values. Authors should also consider reporting actual p-values for significant data (instead of indicating p<0.0001).**

We modified this point in the new manuscript.

**• This sentence is awkwardly written: “In our previous published study 4, we demonstrated that this strategy led to the diagnosis of sABMR in up to 40 %.”**

We completely agree with your comment. We modified this sentence in the text.

**• The discussion should be condensed to be more concise and direct because it lacks clear focus. The discussion should also better delineate between the findings from the group’s previous work and the findings form the current study. Finally, authors should be cautious about describing data as “perfectly comparable” to previous studies.**

Thank you for this comment. We modified the discussion in order to be more concise and direct. We also tried to delineate between the findings from our group’s previous work and the findings form the current study.

**• “Finally” is spelled incorrectly in the discussion.**

We modified this point in the new manuscript.
